# Phenyl 4-methyl­benzoate

**DOI:** 10.1107/S1600536809039361

**Published:** 2009-10-03

**Authors:** B. Thimme Gowda, Miroslav Tokarčík, Jozef Kožíšek, P. A. Suchetan, Hartmut Fuess

**Affiliations:** a Department of Chemistry, Mangalore University, Mangalagangotri 574 199, Mangalore, India; b Faculty of Chemical and Food Technology, Slovak Technical University, Radlinského 9, SK-812 37 Bratislava, Slovak Republic; c Institute of Materials Science, Darmstadt University of Technology, Petersenstrasse 23, D-64287 Darmstadt, Germany

## Abstract

The structure of the title compound, C_14_H_12_O_2_, resembles those of phenyl benzoate and 4-methyl­phenyl benzoate, with similar bond parameters. The two aromatic rings make a dihedral angle of 76.0 (1)°. The plane of the central —C(=O)—O— group is twisted by 9.4 (2)° out of the plane of the benzoyl ring, and by 83.3 (1)° out of the plane of the phenyl ring. The crystal structure exhibits weak parallel stacking of the benzoyl rings, with an inter­planar distance of 3.65 Å and an offset of 1.84 Å. The methyl group shows orientational disorder.

## Related literature

For preparation of the compound, see: Nayak & Gowda (2009 ▶). For background to our study of the effects of substituents on the crystal structures of aryl benzoates and for related structures, see: Gowda *et al.* (2007*a* ▶,*b* ▶, 2008 ▶). For phen­yl benzoate, see: Adams & Morsi (1976 ▶);
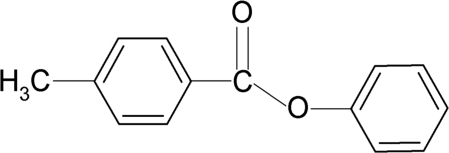


## Experimental

### 

#### Crystal data

C_14_H_12_O_2_
*M* *_r_* = 212.24
Monoclinic, 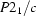

*a* = 12.3440 (4) Å
*b* = 8.1332 (2) Å
*c* = 12.1545 (4) Å
β = 110.911 (4)°
*V* = 1139.89 (6) Å^3^
*Z* = 4
Mo *K*α radiation
μ = 0.08 mm^−1^
*T* = 295 K
0.52 × 0.46 × 0.32 mm

#### Data collection

Oxford Diffraction Xcalibur diffractometer with a Ruby (Gemini Mo) detector
Absorption correction: multi-scan (*CrysAlis RED*; Oxford Diffraction, 2009 ▶) *T*_min_ = 0.96, *T*_max_ = 0.98
20946 measured reflections
2138 independent reflections
1468 reflections with *I* > 2σ(*I*)
*R*_int_ = 0.027

#### Refinement

*R*[*F* ^2^ > 2σ(*F* ^2^)] = 0.046
*wR*(*F* ^2^) = 0.145
*S* = 1.03
2138 reflections
146 parameters
H-atom parameters constrained
Δρ_max_ = 0.23 e Å^−3^
Δρ_min_ = −0.16 e Å^−3^

### 

Data collection: *CrysAlis CCD* (Oxford Diffraction, 2009 ▶); cell refinement: *CrysAlis RED* (Oxford Diffraction , 2009 ▶); data reduction: *CrysAlis RED*; program(s) used to solve structure: *SHELXS97* (Sheldrick, 2008 ▶); program(s) used to refine structure: *SHELXL97* (Sheldrick, 2008 ▶); molecular graphics: *ORTEP-3* (Farrugia, 1997 ▶) and *DIAMOND* (Brandenburg, 2002 ▶); software used to prepare material for publication: *SHELXL97*, *PLATON* (Spek, 2009 ▶) and *WinGX* (Farrugia, 1999 ▶).

## Supplementary Material

Crystal structure: contains datablocks I, global. DOI: 10.1107/S1600536809039361/om2279sup1.cif

Structure factors: contains datablocks I. DOI: 10.1107/S1600536809039361/om2279Isup2.hkl

Additional supplementary materials:  crystallographic information; 3D view; checkCIF report

## Figures and Tables

**Fig. 1.**
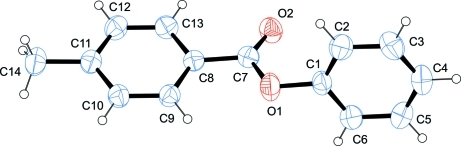
Molecular structure of the title compound showing the atom labelling scheme. Displacement ellipsoids are drawn at the 30% probability level and H atoms are represented as small spheres of arbitrary radii.

**Fig. 2.**
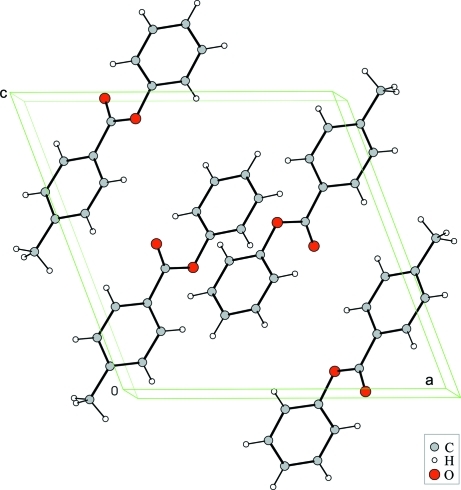
Molecular packing of the title compound.

## supplementary crystallographic information

### Comment

In the present work, as a part of the study of the substituent effects on the crystal structures of aryl benzoates (Gowda *et al.*, 2007*a*, *b*; 2008), the structure of phenyl-4-methylbenzoate (I) has been determined. The structure of (I) (Fig. 1) is similar to those of phenyl benzoate (II) (Adams & Morsi, 1976), 4-methylphenyl benzoate (III) (Gowda *et al.*, 2007*b*), 4-methylphenyl 2-methylbenzoate (IV) (Gowda *et al.*, 2008), 4-methylphenyl 4-methylbenzoate (V) (Gowda *et al.*, 2007*a*) and other aryl benzoates. The two benzene rings make a dihedral angle of 76.0 (1)°, compared to the values of 55.7° for (II), 60.17 (7)° (III), 73.04 (8)° (IV) and 63.57 (5)° (V). The plane of the central –C(=O)–O– group in (I) is twisted 9.4 (2)° out of the plane of the benzoyl ring, and 83.3 (1)° out of the plane of the phenyl ring. The crystal structure exhibits weak parallel stacking of benzoyl rings, with interplanar distance of 3.65 Å and offset 1.84 Å. In the crystal structure, there are no classical hydrogen bonds. The molecules in the structure are packed into chains as viewed in the *ac* plane (Fig. 2).

### Experimental

The title compound was prepared according to a literature method (Nayak & Gowda, 2009). The purity of the compound was checked by determination of its melting point. It was characterized by infrared and NMR spectra (Nayak & Gowda, 2009). Colorless single crystals of the title compound were obtained by slow evaporation of its ethanol solution.

### Refinement

All hydrogen atoms were placed in calculated positions with C–H distances 0.93 or 0.96 Å. The C14 methyl group shows orientational disorder in the hydrogen atom positions. The two sets of methyl hydrogen atoms were refined with equal occupancy. The *U*_iso_(H) values were set at 1.2 *U*_eq_(C-aromatic) or 1.5 *U*_eq_(*C*-methyl).

### Crystal data

**Table:**

C_14_H_12_O_2_	*F*(000) = 448
*M**_r_* = 212.24	*D*_x_ = 1.237 Mg m^−^^3^
Monoclinic, *P*2_1_/*c*	Mo *K*α radiation, λ = 0.71073 Å
Hall symbol: -P 2ybc	Cell parameters from 9248 reflections
*a* = 12.3440 (4) Å	θ = 3.1–29.3°
*b* = 8.1332 (2) Å	µ = 0.08 mm^−^^1^
*c* = 12.1545 (4) Å	*T* = 295 K
β = 110.911 (4)°	Block, colourless
*V* = 1139.89 (6) Å^3^	0.52 × 0.46 × 0.32 mm
*Z* = 4	

### Data collection

**Table:**

Oxford Diffraction Xcalibur diffractometer with a Ruby (Gemini Mo) detector	2138 independent reflections
graphite	1468 reflections with *I* > 2σ(*I*)
Detector resolution: 10.434 pixels mm^-1^	*R*_int_ = 0.027
ω scans	θ_max_ = 25.6°, θ_min_ = 3.1°
Absorption correction: multi-scan (*CrysAlis RED*; Oxford Diffraction, 2009)	*h* = −15→15
*T*_min_ = 0.96, *T*_max_ = 0.98	*k* = −9→9
20946 measured reflections	*l* = −14→14

### Refinement

**Table:**

Refinement on *F*^2^	Primary atom site location: structure-invariant direct methods
Least-squares matrix: full	Secondary atom site location: difference Fourier map
*R*[*F*^2^ > 2σ(*F*^2^)] = 0.046	Hydrogen site location: inferred from neighbouring sites
*wR*(*F*^2^) = 0.145	H-atom parameters constrained
*S* = 1.03	*w* = 1/[σ^2^(*F*_o_^2^) + (0.0889*P*)^2^ + 0.0575*P*] where *P* = (*F*_o_^2^ + 2*F*_c_^2^)/3
2138 reflections	(Δ/σ)_max_ = 0.001
146 parameters	Δρ_max_ = 0.23 e Å^−^^3^
0 restraints	Δρ_min_ = −0.15 e Å^−^^3^

### Special details

**Table:**

Geometry. All e.s.d.'s (except the e.s.d. in the dihedral angle between two l.s. planes) are estimated using the full covariance matrix. The cell e.s.d.'s are taken into account individually in the estimation of e.s.d.'s in distances, angles and torsion angles; correlations between e.s.d.'s in cell parameters are only used when they are defined by crystal symmetry. An approximate (isotropic) treatment of cell e.s.d.'s is used for estimating e.s.d.'s involving l.s. planes.
Refinement. Refinement of *F*^2^ against ALL reflections. The weighted *R*-factor *wR* and goodness of fit *S* are based on *F*^2^, conventional *R*-factors *R* are based on *F*, with *F* set to zero for negative *F*^2^. The threshold expression of *F*^2^ > σ(*F*^2^) is used only for calculating *R*-factors(gt) *etc*. and is not relevant to the choice of reflections for refinement. *R*-factors based on *F*^2^ are statistically about twice as large as those based on *F*, and *R*- factors based on ALL data will be even larger.

### Fractional atomic coordinates and isotropic or equivalent isotropic displacement parameters (Å^2^)

**Table:**

	*x*	*y*	*z*	*U*_iso_*/*U*_eq_	Occ. (<1)
C1	0.44050 (14)	0.7055 (2)	0.52718 (13)	0.0649 (4)	
C2	0.42337 (16)	0.6054 (2)	0.60914 (16)	0.0826 (5)	
H2	0.3509	0.559	0.5955	0.099*	
C3	0.51407 (17)	0.5731 (2)	0.71233 (15)	0.0847 (6)	
H3	0.503	0.5045	0.7686	0.102*	
C4	0.61978 (16)	0.6415 (2)	0.73202 (15)	0.0809 (5)	
H4	0.681	0.6201	0.8019	0.097*	
C5	0.63602 (14)	0.7415 (2)	0.64933 (16)	0.0792 (5)	
H5	0.7085	0.7882	0.6634	0.095*	
C6	0.54637 (15)	0.7745 (2)	0.54505 (15)	0.0737 (5)	
H6	0.5577	0.8419	0.4883	0.088*	
C7	0.26747 (13)	0.84141 (18)	0.41132 (13)	0.0602 (4)	
C8	0.17152 (12)	0.83643 (17)	0.29559 (12)	0.0558 (4)	
C9	0.17601 (13)	0.74161 (18)	0.20301 (13)	0.0629 (4)	
H9	0.2427	0.6822	0.2108	0.075*	
C10	0.08196 (13)	0.73479 (19)	0.09922 (14)	0.0660 (4)	
H10	0.0868	0.6716	0.0374	0.079*	
C11	−0.01947 (13)	0.81929 (18)	0.08448 (13)	0.0633 (4)	
C12	−0.02227 (14)	0.9156 (2)	0.17716 (15)	0.0718 (5)	
H12	−0.0889	0.9754	0.1691	0.086*	
C13	0.07132 (14)	0.92496 (18)	0.28101 (14)	0.0679 (4)	
H13	0.0674	0.9911	0.3419	0.082*	
C14	−0.12267 (15)	0.8048 (2)	−0.02748 (15)	0.0835 (5)	
H14A	−0.1656	0.906	−0.042	0.125*	0.5
H14B	−0.1715	0.7167	−0.0203	0.125*	0.5
H14C	−0.097	0.7825	−0.0918	0.125*	0.5
H14D	−0.1924	0.8088	−0.0098	0.125*	0.5
H14E	−0.1192	0.7023	−0.0652	0.125*	0.5
H14F	−0.1224	0.8941	−0.0791	0.125*	0.5
O1	0.35063 (10)	0.72961 (15)	0.41782 (10)	0.0865 (4)	
O2	0.27223 (9)	0.92969 (13)	0.49154 (9)	0.0758 (4)	

### Atomic displacement parameters (Å^2^)

**Table:**

	*U*^11^	*U*^22^	*U*^33^	*U*^12^	*U*^13^	*U*^23^
C1	0.0612 (9)	0.0712 (10)	0.0534 (8)	0.0089 (8)	0.0095 (7)	−0.0108 (7)
C2	0.0734 (11)	0.0913 (12)	0.0790 (11)	−0.0181 (9)	0.0221 (9)	−0.0136 (10)
C3	0.1043 (15)	0.0767 (11)	0.0670 (11)	−0.0104 (10)	0.0230 (10)	0.0040 (8)
C4	0.0817 (12)	0.0714 (10)	0.0689 (11)	0.0066 (9)	0.0016 (9)	−0.0025 (8)
C5	0.0599 (10)	0.0815 (12)	0.0842 (12)	−0.0029 (8)	0.0110 (9)	−0.0022 (9)
C6	0.0729 (11)	0.0763 (11)	0.0705 (10)	0.0036 (9)	0.0238 (9)	0.0047 (8)
C7	0.0624 (9)	0.0576 (8)	0.0608 (9)	−0.0016 (7)	0.0222 (7)	−0.0014 (7)
C8	0.0562 (8)	0.0526 (8)	0.0581 (8)	−0.0029 (6)	0.0198 (7)	0.0001 (6)
C9	0.0572 (9)	0.0656 (9)	0.0629 (9)	0.0044 (7)	0.0178 (7)	−0.0038 (7)
C10	0.0642 (10)	0.0704 (10)	0.0597 (9)	−0.0018 (8)	0.0176 (8)	−0.0059 (7)
C11	0.0589 (9)	0.0602 (9)	0.0651 (9)	−0.0043 (7)	0.0153 (7)	0.0109 (7)
C12	0.0622 (10)	0.0683 (10)	0.0806 (11)	0.0143 (8)	0.0204 (8)	0.0094 (8)
C13	0.0733 (10)	0.0615 (9)	0.0697 (10)	0.0077 (8)	0.0264 (8)	−0.0012 (7)
C14	0.0669 (10)	0.0899 (12)	0.0778 (11)	−0.0072 (9)	0.0063 (9)	0.0131 (9)
O1	0.0739 (8)	0.1056 (9)	0.0623 (7)	0.0271 (7)	0.0024 (6)	−0.0195 (6)
O2	0.0821 (8)	0.0734 (7)	0.0660 (7)	0.0022 (6)	0.0193 (6)	−0.0148 (5)

### Geometric parameters (Å, °)

**Table:**

C1—C2	1.361 (2)	C8—C13	1.387 (2)
C1—C6	1.366 (2)	C9—C10	1.377 (2)
C1—O1	1.4082 (18)	C9—H9	0.93
C2—C3	1.376 (2)	C10—C11	1.383 (2)
C2—H2	0.93	C10—H10	0.93
C3—C4	1.359 (2)	C11—C12	1.383 (2)
C3—H3	0.93	C11—C14	1.500 (2)
C4—C5	1.362 (2)	C12—C13	1.376 (2)
C4—H4	0.93	C12—H12	0.93
C5—C6	1.379 (2)	C13—H13	0.93
C5—H5	0.93	C14—H14A	0.96
C6—H6	0.93	C14—H14B	0.96
C7—O2	1.1954 (16)	C14—H14C	0.96
C7—O1	1.3524 (18)	C14—H14D	0.96
C7—C8	1.481 (2)	C14—H14E	0.96
C8—C9	1.381 (2)	C14—H14F	0.96
			
C2—C1—C6	121.27 (15)	C8—C9—H9	119.9
C2—C1—O1	119.90 (15)	C9—C10—C11	121.80 (14)
C6—C1—O1	118.65 (15)	C9—C10—H10	119.1
C1—C2—C3	119.54 (17)	C11—C10—H10	119.1
C1—C2—H2	120.2	C12—C11—C10	117.44 (14)
C3—C2—H2	120.2	C12—C11—C14	121.56 (15)
C4—C3—C2	120.02 (17)	C10—C11—C14	121.00 (15)
C4—C3—H3	120	C13—C12—C11	121.45 (15)
C2—C3—H3	120	C13—C12—H12	119.3
C3—C4—C5	119.96 (16)	C11—C12—H12	119.3
C3—C4—H4	120	C12—C13—C8	120.48 (15)
C5—C4—H4	120	C12—C13—H13	119.8
C4—C5—C6	120.86 (17)	C8—C13—H13	119.8
C4—C5—H5	119.6	C11—C14—H14A	109.5
C6—C5—H5	119.6	C11—C14—H14B	109.5
C1—C6—C5	118.34 (16)	H14A—C14—H14B	109.5
C1—C6—H6	120.8	C11—C14—H14C	109.5
C5—C6—H6	120.8	H14A—C14—H14C	109.5
O2—C7—O1	122.75 (13)	H14B—C14—H14C	109.5
O2—C7—C8	125.51 (14)	C11—C14—H14D	109.5
O1—C7—C8	111.72 (12)	C11—C14—H14E	109.5
C9—C8—C13	118.62 (14)	H14D—C14—H14E	109.5
C9—C8—C7	122.54 (13)	C11—C14—H14F	109.5
C13—C8—C7	118.79 (13)	H14D—C14—H14F	109.5
C10—C9—C8	120.18 (14)	H14E—C14—H14F	109.5
C10—C9—H9	119.9	C7—O1—C1	118.32 (11)
			
C6—C1—C2—C3	0.3 (3)	C7—C8—C9—C10	176.63 (14)
O1—C1—C2—C3	175.36 (15)	C8—C9—C10—C11	−0.9 (2)
C1—C2—C3—C4	0.2 (3)	C9—C10—C11—C12	1.8 (2)
C2—C3—C4—C5	−0.3 (3)	C9—C10—C11—C14	−177.47 (14)
C3—C4—C5—C6	−0.1 (3)	C10—C11—C12—C13	−1.2 (2)
C2—C1—C6—C5	−0.7 (3)	C14—C11—C12—C13	178.09 (15)
O1—C1—C6—C5	−175.82 (14)	C11—C12—C13—C8	−0.3 (2)
C4—C5—C6—C1	0.6 (3)	C9—C8—C13—C12	1.3 (2)
O2—C7—C8—C9	174.22 (14)	C7—C8—C13—C12	−176.12 (14)
O1—C7—C8—C9	−7.1 (2)	O2—C7—O1—C1	6.3 (2)
O2—C7—C8—C13	−8.5 (2)	C8—C7—O1—C1	−172.34 (13)
O1—C7—C8—C13	170.12 (13)	C2—C1—O1—C7	82.15 (19)
C13—C8—C9—C10	−0.6 (2)	C6—C1—O1—C7	−102.63 (18)
